# Editorial: Case reports in heart failure and transplantation: 2022

**DOI:** 10.3389/fcvm.2023.1282064

**Published:** 2023-09-11

**Authors:** Matteo Cameli, Federico Landra

**Affiliations:** Department of Medical Biotechnologies, Division of Cardiology, University of Siena, Siena, Italy

**Keywords:** heart failure, heart transplant, takotsubo, genetic, devices, advanced heart failure

## Abstract

In this editorial we summarize the most viewed and downloaded contributing articles to the Research Topic “Case Reports in Heart Failure and Transplantation: 2022” of the journal Frontiers in Cardiovascular Medicine.

**Editorial on the Research Topic** Case reports in heart failure and transplantation: 2022

## The adjunctive value of genetic testing

Even though it may be difficult to glean interpretable results in the absence of a specific clinical suspicion, nowadays genetic testing has largely established as a fundamental tool both in the diagnostic workflow and risk stratification of a great variety of cardiac diseases. A similar revolution in the cardiovascular field has been recognized with the introduction of cardiac magnetic resonance, which brought the possibility to unveil structural abnormalities without the need of a histological specimen. However, tissue alterations are only the epiphenomenon of an underlying pathological process and we sometimes fail to go beyond the ultrastructural understanding. In this context, genetic testing may help identifying the missing link piece, finally leading to a comprehensive characterization of the pathological process. This is the case of the patient reported by Liu X et al. which eventually appeared to be affected by a rare desmin myopathy. Diagnosis was only possible with the aid of genetic testing, which identified a missense mutation (c.1366G > A) in the desmin gene, and immunohistochemical staining confirmed desmin deficiency. Clearly, we are still far from tailored therapies for each genetic disease, so one could argue what is the adjunctive value of such pathological understanding. Heart failure and arrhythmia treatments would have been initiated irrespective of the diagnosis of desmin myopathy. However, being able to call a pathological entity by its name and its genetic underpinning give us the chance not only to focus more specifically on its peculiar characteristics, such as, for instance, conduction disturbances occurrence in the case of desmin myopathy, but also to diagnose the disease in a preclinical form in patient's relatives. In addition, the well-known advent of the precision medicine can cast the light of hope on the future of these progressive diseases.

## Increasing knowledge in Takotsubo cardiomyopathy pathophysiology

Recently, growing evidence is forming around the mysterious pathophysiology of Takotsubo stress cardiomyopathy. As a proof of that, only in this Research Topic as much as three articles out of seventeen take into account this specific nosological entity. Takotsubo is known to be triggered by emotional or physical stress, and acute cathecolamine release is considered to be the causative event leading to cardiac dysfunction. Wei et al. presented an interesting case of an iatrogenic Takotsubo cardiomyopathy, occurred after injection of high doses of epinephrine infusion for cardiopulmonary resuscitation. This case confirms the central role of cathecolamines and poses the problem of treating those patients presenting with cardiogenic shock, since the much needed cathecolamines may paradoxically deteriorate the hemodynamic status. In contrast, beta blockers are usually beneficial, but overt heart failure is a clear contraindication, and levosimendan may be a reasonable option. Lastly, a potential common pathological substrate has been reported between cancer and Takotsubo cardiomyopathy, namely an individually high catecholaminergic state, underlying the need for careful investigation in that sense.

In addition to catecholamine excess, the pathogenesis could be linked also to other factors such as inflammatory responses and endothelial dysfunctions. Gudenkauf et al. nicely described a case of cytokine storm triggering Takotsubo cardiomyopathy. Cytokine storm is a clinically diagnosed condition presenting with fever, absence of signs of infections and elevated inflammatory biomarkers. In this case, cytokine storm was also associated with rapid development of lactic acidosis and hemodynamic compromise. Even though treatment of cytokine storm has not been clearly defined so far, and much interest is directed towards molecules specifically targeting single cytokines, the Authors showed the beneficial role of pulse dose steroids for the treatment of inflammation associated with Takotsubo syndrome. Further research will clarify in more detail an integrated pathophysiological process and better characterize treatment rationale and implementation depending on the specific phenotype of the disease.

## New device options for advanced heart failure

We are now familiar with the implementation of devices in the treatment of heart failure, ranging from cardiac resynchronization therapy to mechanical circulatory support systems such as left ventricular assist devices. The improvements in medical and device therapy for chronic heart failure are dramatically increasing the number of patients worldwide living with this disease. A significant portion of these patients face a progressive decline in heart function and functional capacity, moving towards an advanced stage of the disease. At this point of the natural history of heart failure, patients may no longer tolerate full dose of medical treatments, therefore prognosis consequently worsen as witnessed by the higher number of heart failure-related hospitalizations. Heart transplant is the most effective treatment for these patients, but strict contraindications and the reduced number of organ availability limit the feasibility for most of the patients with advanced heart failure. Fortunately, many device therapies are available and play a major role at this stage, bringing prognostic benefit and improving quality of life. Cardiac contractility modulation is a novel device-based therapy for patients with heart failure with reduced ejection fraction and may be considered a valuable treatment option also for those patients in advanced stage in whom no other therapeutic strategies may be pursued. Cardiac contractility modulation has been shown to improve functional class, reverse left ventricular remodeling and reduce hospitalization in patients with reduced ejection fraction. In the case presented by Masarone et al. cardiac contractility modulation has been successfully used as a bridge to transplant strategy for an obese patient in whom no other viable options were available. In the follow-up, patient's quality of life improved, along with reduction of left ventricular filling pressure and level of N-terminal prohormone of brain natriuretic peptide. In another case by Visco et al. the hemodynamic improvement achieved by cardiac contractility modulation was elegantly demonstrated by data collected from the CardioMEMS device. CardioMEMS is another device which may be used for symptomatic patients with heart failure and recent hospitalization, placed at pulmonary artery level with the purpose of monitoring cardiac filling pressure. Having an invasive hemodynamic monitoring system allows prompt identification of congestion and treatment. In this case, for the first time the usefulness of cardiac contractility modulation was objectively proven by continuous invasive monitoring of pulmonary arterial pressures.

## Final considerations

Generally speaking, research has the role to improve both diseases understanding and diseases treatment. This Research topic homogeneously included case reports which brought additional insights into these two directions. Aside from the papers highlighted herein, many other high-quality works have been published in this topic which well deserve a lecture. From heart transplant-related infections to rare cardiomyopathies, from mechanical circulatory support to cardiac rehabilitation, this series of clinical cases spans over a wide range of subjects concerning heart failure.

